# Integrated Haematological Profiles of Redox *Status*, Lipid, and Inflammatory Protein Biomarkers in Benign Obesity and Unhealthy Obesity with Metabolic Syndrome

**DOI:** 10.1155/2015/490613

**Published:** 2015-05-18

**Authors:** Carla Lubrano, Giuseppe Valacchi, Palma Specchia, Lucio Gnessi, Elizaveta P. Rubanenko, Elena A. Shuginina, Arseny I. Trukhanov, Liudmila G. Korkina, Chiara De Luca

**Affiliations:** ^1^Section of Medical Pathophysiology, Endocrinology and Food Science, Department of Experimental Medicine, “Sapienza” University, “Umberto I” Polyclinic, Viale Regina Elena 324, 00161 Rome, Italy; ^2^Department of Life Sciences and Biotechnology, University of Ferrara, Via Luigi Borsari 46, 44100 Ferrara, Italy; ^3^Department of Food and Nutrition, Kyung Hee University, 26 Kyungheedae-ro, Dongdaemun-gu, Seoul 130-701, Republic of Korea; ^4^Active Longevity Clinic “Institut Krasoty na Arbate”, 8 Maly Nikolopeskovsky Lane, Moscow 119002, Russia; ^5^Centre of Innovative Biotechnological Investigations (Cibi-NanoLab), 197 Vernadskogo Prospekt, Moscow 119571, Russia

## Abstract

The pathogenesis of obesity (OB) and metabolic syndrome (MetS) implies free radical-, oxidized lipid- (LOOH-), and inflammatory cytokine-mediated altered pathways in target organs. Key elements of the transition from benign OB to unhealthy OB+MetS remain unclear. Here, we measured a panel of redox, antioxidant, and inflammation markers in the groups of OB patients (67 with, 45 without MetS) and 90 controls. Both OB groups displayed elevated levels of adipokines and heavy oxidative stress (OS) evidenced by reduced levels of glutathione, downregulated glutathione-S-transferase, increased 4-hydroxynonenal-protein adducts, reactive oxygen species, and membrane-bound monounsaturated fatty acids (MUFA). Exclusively in OB+MetS, higher-than-normal glutathione peroxidase activity, tumor necrosis factor-*α*, and other proinflammatory cytokines/chemokines/growth factors were observed; a combination of high adipokine plasminogen activator inhibitor-1 and MUFA was consistent with increased cardiovascular risk. The uncomplicated OB group showed features of adaptation to OS such as decreased levels of vitamin E, activated superoxide dismutase, and inhibited catalase, suggesting H_2_O_2_ hyperproduction. Proinflammatory cytokine pattern was normal, except few markers like RANTES, a suitable candidate for therapeutic approaches to prevent a setting of MetS by inhibition of LOOH-primed leukocyte chemotaxis/recruitment to target tissues.

## 1. Introduction

The constant growth of obesity pandemic has fostered the research of reliable biomarkers for routine diagnosis, able to assess the severity of the underlying metabolic impairment, to predict risk of the wide array of multiorgan complications, and to provide solid molecular basis for innovative therapeutic approaches [1–3]. Distinctive ties between metabolic dysfunction and immune system alterations have also produced relevant achievements, leading to the classification of the adipose tissue as an effective immunoendocrinological organ [4–6]. Metabolic syndrome (MetS) is the most distinctive condition featuring “nonbenign obesity” (morbid obesity) [7, 8], which encompasses the health-impairing forms of obesity [9–11]. The different criteria are adopted worldwide, also on ethnic basis, to diagnose MetS; all include the key parameters of fasting plasma glycemia, total and HDL cholesterol levels, fasting triglyceride levels, blood pressure, waist circumference, body mass index (BMI), and insulin resistance [7, 12–15].

Various combinations of these altered parameters have been unequivocally related to the individual higher risk of endocrinological [16, 17], hepatic [18, 19], cardiovascular [20, 21], neurological [22, 23], and ultimately oncological [24] outcomes. More recently the immunological state, namely, the altered transcription levels and/or the altered concentrations levels of several systemic proinflammatory cytokines, chemokines, and growth factors, as well as adipose-tissue-specific adipokines, have gained focus as key parameters driving mere overweight into a MetS [25]. Their alterations have been directly correlated with lipid metabolism, adipogenesis and fat distribution, adipocyte metabolism and function, glucose uptake abnormalities, insulin secretion and resistance, appetite and satiety, energy expenditure, endothelial function, hemostasis, blood pressure, energy metabolism in insulin sensitive tissues, and immune cell migration into adipose tissue [26–30].

The role of systemic redox state and of antioxidant (AO) imbalance has also been studied in obesity in terms of molecular mechanisms of disease (for recent review see [31]) and of nutritional intervention protocols aimed at weight control and normalization of metabolic parameters (for review see [32]). In particular, the etiological relevance of excessive reactive-species-mediated oxidation of lipids, carbohydrates, and proteins in the different organs targeted by MetS has been extensively described [33–35]. The current diagnostic guidelines of MetS and morbid obesity are essentially based on type II diabetes protocols and traditionally employ combined approaches, that is, the anthropometric profile (height, weight, waist/hip/arm circumference, etc.), mainly reported as BMI index [36], coupled with the assessment of clinical laboratory parameters connected with glucose and lipid metabolism [7, 12–15], hormone status [12], routine protein markers of inflammation [37], bone, hepatic, and renal markers [38, 39]. The reliability of BMI and waist circumference parameters is currently being discussed, as unable to correlate with the individual levels of lipid and glucose dysmetabolism or of chronic inflammation [40, 41]. Overcoming biopsy invasiveness, technology-assisted noninvasive evaluation methods of fat and lean mass, of liver or cardiac fat, and so forth have been introduced as additional clinical parameters correlating with severity of obesity [39, 42]. Conversely, the laboratory blood test protocols for MetS and obesity have not been updated with clinical research outcomes. Thus far, neither the proinflammatory cytokines and adipokines panels, in effect requiring elevated costs, nor the redox parameters, more easily measurable on a routine basis, have been examined extensively in the different obese types.

In the attempt to contribute to the ongoing efforts for the identification of new pathologically relevant molecular markers of obese morbidity, we attempted here to identify possible significative alterations of a panel of 55 metabolic blood markers of redox* status*, nutritional and endogenous AO/free radical-detoxifying defenses, and unsaturated lipid quality and oxidation grade, in a group of 112 Italian Caucasian obese patients, of which 67 with obese state aggravated by MetS, as compared with a control group of 90 healthy subjects. In addition, the concurrent analysis of a panel of 37 serum cytokines and adipokines enabled a comparative evaluation of the chronic proinflammatory levels in the two obesity subgroups. Ultimately, the study protocol aimed at identifying possible specific redox-related metabolic and immunologic patterns marking the transition from a state of “healthy” obesity to morbid obesity with MetS and increased type II diabetes risk. The selection of a specific panel of reliable molecular determinants for this transition bears promising implications for diagnosis, risk prevention, and follow-up of obese subjects with different degrees of impaired metabolism and possibly will provide new hints for future treatment innovation.

## 2. Materials and Methods

### 2.1. Patients

The study enrolled a group of 112 Italian Caucasian obese subjects at “Umberto I” Polyclinic, and IDI IRCCS-Istituto Dermopatico dell'Immacolata, Rome, Italy. The patients, included on the basis of their consecutive attendance to the obesity outpatient facilities of the two centers, displayed the following sex distribution: 100 females (89%) and 12 males (11%). The study protocol was reviewed and approved by the hospital Ethical Committee Board IDI IRCCS n.121/CE/2008. Of the patient group, *n* = 67 subjects were diagnosed with metabolic syndrome (OB w.MetS, 59F/8M, age: m ± S.D. = 44.4 ± 12.1) and *n* = 45 did not meet MetS criteria (OB w.o.MetS, 41F/4M, age: m ± S.D. = 42.6 ± 11.7), as determined according to revised NCEP ATP III (US National Cholesterol Education Program, Adult Treatment Panel) criteria, cut-off value for abdominal obesity 94 cm for men and 80 cm for women [12]. Patients with overt endocrine pathology, acute illnesses, heart diseases, uncontrolled hypertension, current use of hypnotics, or any treatment for breathing disorders were excluded from enrollment.

A cohort of 90 healthy age- and sex-matched subjects were enrolled as the control group (CTR, age m ± S.D. = 42.5 ± 11.7),  83 females (92%) and 7 males (study protocol approval by IDI IRCCS Ethical Committee, n.52/CE/2010), according to the established criteria of (i) absence of any clinically diagnosed disease, in particular allergic or immunologic disturbances and (ii) whole blood total production of reactive oxygen and nitrogen species (ROS/RNS) below 650 cps/*μ*L, as determined by luminol-dependent chemiluminescent response to phorbol 12-myristate 13-acetate (PMA) [43].

No patients/controls entering the study had taken any drugs or nutraceutical supplements known to interfere with metabolizing/AO enzymes activity since at least six weeks, at the time of blood sampling. No alcohol- or drug-abusers were present in any of the three cohorts studied. All subjects gave their informed consent to personal and anamnestic data collection, blood sampling for the specific sets of analyses, and blood fractions banking. Anthropometric data and detailed clinical history were registered by trained medical personnel. Routine blood parameters examined in the obese patient including fasting total cholesterol (CHOL), low density lipoproteins (LDL), high density lipoproteins (HDL), triglycerides (TG), glucose, insulin, C-reactive protein (CRP), alkaline phosphatase (ALP), aspartate aminotransferase (AST), alanine aminotransferase (ALT), gamma glutamyltransferase (gamma GT) were evaluated with routine clinical chemistry laboratory methods. BMI was calculated as weight in kg divided by the square of height in meters (kg/m^2^) [44].

Fat mass was measured by dual energy X-ray absorptiometry (DEXA) (Hologic Inc., Bedford, MA, USA, QDR 4500 W, S/N 47168) [42] by one single experienced technician. Specific delimiters for regional analysis were determined by standard software (Hologic Inc., QDR 4500 W S/N 47168 VER. 11.2). Total body fat mass was expressed in percentage of total weight.

### 2.2. Reagents and Assay Kits

Majority of chemical reagents, HPLC standards, mediums, and fluorogenic probes were from Sigma Chemical Co. (St. Louis, MO, USA); kits for enzyme activity assays were from Cayman Chem. Co. (Ann Arbor, MI, USA); Western blot reagents and antibodies were from Invitrogen (Milan, Italy), Millipore (Billerica, MA), Santa Cruz Biotechnology, Inc. (Santa Cruz, CA), Bio-Rad Laboratories, Inc. (Hercules, CA, USA), and Amersham Biosciences (Milan, Italy).

### 2.3. Redox and Oxidation Marker Studies

Complete differential blood cell counts and metabolic analyses were performed on fresh ethylenediaminetetraacetic acid (EDTA)-anticoagulated venous blood of 12 hr-fasting subjects. Biochemical assays were performed on peripheral blood plasma or erythrocytes (RBC) either immediately (ATP, glutathione, and coenzyme Q_10_) or within 72 hr on sample aliquots stored at −80°C under argon. Whole blood luminol-dependent chemiluminescence (CL, expressed in counts per second, cps/*μ*L) response to PMA was quantified by a Victor^2^ 1420 multilabel counter, equipped with Wallac 1420 software (Perkin Elmer, MA, USA) according to [43]; levels of nitrites/nitrates (NO_2_^−^/NO_3_^−^, expressed as *μ*mol/L) were measured spectrophotometrically by Griess reagent [45]. Protein content was measured according to Bradford, using Bio-Rad microplate assay kit. Plasmatic total antioxidant capacity (TAC, nmol/L) was determined spectrophotometrically according to [46]. Reduced and oxidized glutathione (GSH, GSSG, mg/L) levels in erythrocytes [47], reduced and oxidized coenzyme Q_10_ (CoQ_10_H_2_, CoQ_10_, *μ*g/L), and *α*-tocopherol (ALPHA-TOC, mg/L) levels in plasma were quantified by HPLC equipped with array photodiode and electrochemical detection as described previously [48]. Activities of CuZn superoxide dismutase (CuZn-SOD, U/g prot. (protein)) [49], catalase (CAT, U/g prot.) [50], glutathione S-transferase (GST, U/mg Hb (haemoglobin)) [51], and glutathione peroxidase (GPX, U/mg Hb) [52] in erythrocytes were measured spectrophotometrically. Differences in 4-hydroxynonenal protein adducts (4-HNE PA) levels in the plasma of obese patients and controls were assessed with semiquantitative approach by Western blot analysis, as previously described [53]. Briefly, plasma samples (60 *μ*g prot.) were diluted in reducing sample buffer and boiled for 10 min. Samples were resolved on a 4–20% sodium dodecyl sulphate-polyacrylamide gradient gel (Invitrogen, Milan, Italy) and electrotransferred onto nitrocellulose membranes. Loading control was performed with ponceau staining. After blocking in 3% fat-free milk in phosphate buffered saline solution, the membranes were incubated overnight at 4°C with goat anti-HNE polyclonal antibodies (Millipore, Billerica, MA) at 1 : 3000 dilution. After washing, mouse anti-goat horseradish peroxidase-conjugated antibodies (Santa Cruz Biotechnology, Inc., Santa Cruz, CA) were used as secondary antibodies. Membranes were processed with the enzymatic chemiluminescence solution (ECL, Immune-Star HRP Substrate Kit, Bio-Rad) and exposed to photographic film (Hyperfilm ECL, Life Science, Amersham Biosciences, Milan, Italy), according to manufacturer recommendations. Band densities were quantified using the National Institute for Health image shareware. Results of three independent experiments were expressed in arbitrary units (usually, corresponding to the *μ*M range), as mean values ± SD.

### 2.4. Erythrocyte Membrane Fatty Acid Profiling

The fatty acid (FA) pattern of erythrocyte membrane phospholipids was analyzed on a subgroup of patients and controls by gas-chromatography coupled with mass spectrometry with the selected ion monitoring technique, set to identify C16:0, C16:1, C18:0, C18:1*cis*, C18:1*trans*, C18:2*ω*6, C18:3*ω*6, C20:4 *ω*6, C20:5*ω*3, C22:4*ω*3, C22:5*ω*3, and C22:6*ω*3 peaks [54]. Results were expressed as percentage of the total fatty acid content of membrane phospholipids for saturated (SAT), mono-unsaturated FA (MUFA), and polyunsaturated FA (PUFA), as percent values of the single representative FA of the *ω*-3- and *ω*-6-PUFA series and as specific ratios, SAT/MUFA and *ω*-6-/*ω*-3-PUFA.

### 2.5. ATP Measurement in Platelet-Enriched Plasma

Adenosine 5′-triphosphate (ATP) levels in activated platelet-enriched plasma (PRP) was measured with a Bioluminescent Assay Kit (Sigma-Aldrich, MO, USA). Fresh EDTA-anticoagulated blood was centrifuged 8 min at 1000 g, and the upper layer without erythrocytes was collected. PRP was kept at room temperature for analysis. Platelets were activated by a 5 min incubation with thrombin. Results were expressed as *μ*mol/L (*μ*M).

### 2.6. Cytokine and Adipokine Plasmatic Profiling

The plasma levels of 27 cytokines, chemokines, and growth factors interleukin, IL-1*β*; IL-1ra; IL-2; IL-4; IL-5; IL-6; IL-7; IL-8; IL-9; IL-10; IL-12; IL-13; IL-15; IL-17; eotaxin; fibroblast growth factor basic, bFGF; granulocyte-colony stimulating factor, G-CSF; granulocyte-macrophage colony stimulating factor, GM-CSF; interferon-*γ*, IFN-gamma; IFN-*γ*-inducible protein 10, IP-10; monocyte chemoattractant protein-1, MCP-1; macrophage inflammatory protein 1-*α*, MIP-1*α*; macrophage inflammatory protein 1-*β*, MIP-1*β* (regulated on activation normal T cell expressed and secreted, RANTES; platelet-derived growth factor-bb, PDGFbb; tumor necrosis factor-*α*, TNF-*α*; vascular endothelial growth factor, VEGF), and 10 adipokines (C-peptide; ghrelin; gastric inhibitory polypeptide, GIP; glucagon-like peptide-1, GLP-1; glucagon; insulin; leptin; plasminogen activator inhibitor-1, PAI-1 total; resistin; visfatin) were measured simultaneously applying multiplexed Bio-Rad assays, using the Bio- Plex Suspension Array System (Bio-Rad Laboratories, Inc., Hercules, CA, USA) [55]. The assay was performed according to the manufacturer's instructions. Cytokine concentrations were expressed in pg/mL of plasma, and each factor was quantified in the linear range of its calibration curve using a Bio-Rad array reader.

### 2.7. Statistical Analysis

Statistical significance of redox and fatty acid parameters was evaluated using STATISTICA 6.0 program (StatSoft Inc., Tulsa, OK, USA). Normality of data was checked using the Shapiro-Wilk test. Since the distribution of the data in the three groups was significantly different from normal, nonparametric statistics were used. Values were presented as mean, standard error of the mean, and 1.96 × standard error of duplicate analyses. Mann-Whitney *U* test for independent samples was employed for comparison between case groups and controls. All reported *p* values are from two-tailed tests, and *p* values of less than 0.05 were considered to indicate statistical significance. If necessary, *p* values were adjusted for multiple comparisons using the Bonferroni adjustment.

## 3. Results

### 3.1. Anamnestic Data

CTR group mean ± SEM values of BMI were 22.5 ± 0.7 kg/m^2^; clinical laboratory parameters fell within normal range (data not shown). Patient self-declared age of obesity onset was 19 ± 2.1 and 17 ± 2.5 yr., and duration of obesity state was 24 ± 2.1 and 19 ± 2.0 yr., respectively, for OB w.MetS and OB w.o.MetS. The main relevant anthropometric and routine clinical laboratory parameters examined by clinicians to set the diagnosis of obesity or of obesity with MetS, are reported in Table 1. According to the World Health Organization definition of obesity classes as a function of BMI [56], obese patients were distributed as follows: 7.8% overweight (BMI = 25.0–29.9), 23.4% class I (BMI = 30.0–34.9), 20.3% class II (BMI = 35.0–39.9), and 48.5% class III (BMI ≥ 40).

### 3.2. RBC Detoxifying and Antioxidant Enzyme Activities

**Figure 1:**
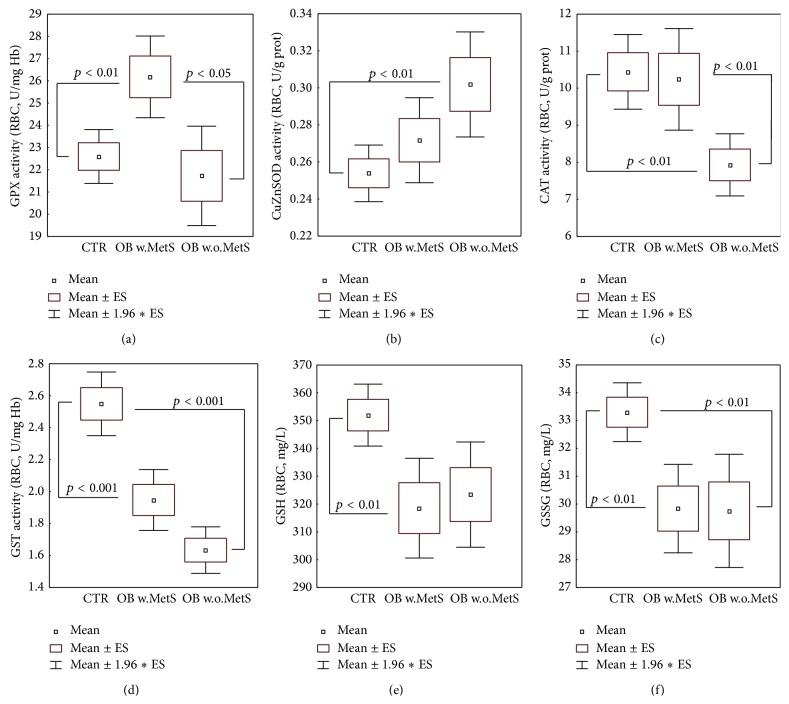
Metabolic redox parameters: erythrocyte levels of antioxidant/detoxifying enzymatic activities of glutathione peroxidase (a), CuZn-superoxide dismutase (b), catalase (c), glutathione-S-transferase (d), and reduced, oxidized glutathione (e-f), in the groups of obese patients with metabolic syndrome (OB w.MetS, *n* = 67) and without metabolic syndrome (OB w.o.MetS, *n* = 45), and of control healthy subjects (CTR, *n* = 90). Values are represented as mean (□), standard error of the mean (upper and lower limits of the box), and 1.96 × standard error (upper and lower whiskers). Intergroup significant differences (*p*) are indicated in each panel. Abbreviations: GPX (glutathione peroxidase), CuZnSOD (CuZn-superoxide dismutase); CAT (catalase); GST (glutathione S-transferase); GSH (reduced glutathione); GSSG (oxidized glutathione); RBC (red blood cells); U (units); prot. (proteins); Hb (haemoglobin). Techniques: spectrophotometric methods (a–d), HPLC with array photodiode detection (e).

Results obtained in the control group and in obese subjects with or without MetS for RBC activities of enzymes committed to the detoxification of oxygen, nitrogen, and oxidized lipid reactive species, as well as to antioxidant functions, are shown in Figure 1. In the OB w.MetS, GPX activity was increased (*p* < 0.01) versus CTR (Figure 1(a)), with correspondent decrease of GSH content in RBC (*p* < 0.01) (Figure 1(e)). In the OB w.o.MetS, no GSH decrease was observed (Figure 1(e)). GPX activity did not result hyperactivated versus CTR and was consistently lower than in the OB w.MetS group (*p* < 0.05) (Figure 1(a)). RBC CuZn-SOD was not elevated in MetS-obese, while it was significantly increased in OB w.o.MetS versus CTR (*p* < 0.01) (Figure 1(b)).  Figure 1(c) shows that CAT activity was significantly suppressed in the same OB w.o.MetS group versus both CTR and OB w.MetS (*p* < 0.01). GST erythrocyte activity (Figure 1(d)) was markedly lower than CTR (*p* < 0.001) in all obese patients. As a whole, obese subgroups suffered a depletion of glutathione erythrocyte levels, affecting both reduced (GSH) and oxidized (GSSG) forms in OB w.MetS (*p* < 0.01) and limited to GSSG in OB w.o.MetS (Figures 1(e) and 1(f)).

### 3.3. Plasmatic Oxidation Markers and Antioxidants

**Figure 2:**
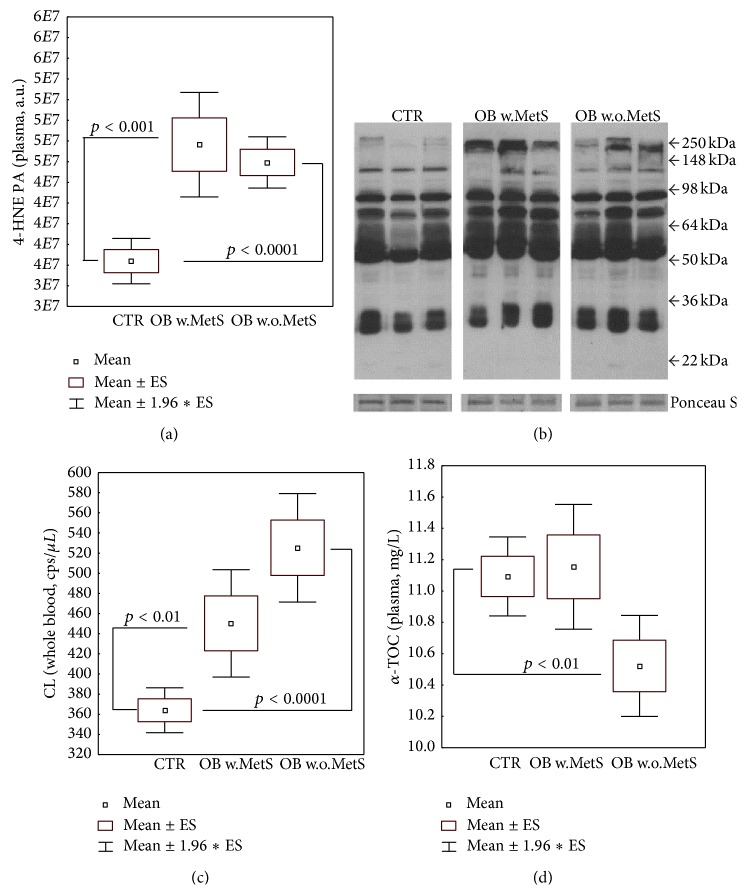
Metabolic redox parameters: levels of plasma 4-hydroxynonenal protein adducts (a) and a representative blot of the relative gel bands of samples and of loading control (ponceau staining) (b), of whole blood luminol-dependent chemiluminescence (c), of plasma *α*-tocopherol (d), in the groups of obese patients with metabolic syndrome (OB w.MetS, *n* = 67) and without metabolic syndrome (OB w.o.MetS, *n* = 45), and of control healthy subjects (CTR, *n* = 90). Values are represented as mean (□), standard error of the mean (upper and lower limits of the box), and 1.96 × standard error (upper and lower whiskers). Intergroup significant differences (*p*) are indicated in each panel. Abbreviations: 4-HNE PA (4-hydroxynonenal protein adducts); kDa (kiloDalton); CL (luminol-dependent chemiluminescence); cps (counts per second); ALPHA-TOC (*α*-tocopherol). Techniques: Western blot (a-b), chemiluminescence (c), and HPLC with array photodiode detection (d).

Consistent with the depletion of the detoxifying cofactor glutathione, 4-HNE PA levels displayed significant elevation in the plasma of both obese cohorts (resp., *p* < 0.001 and *p* < 0.0001, resp., in OB w.MetS and OB w.o.MetS), as compared to healthy normal-weight subjects (Figure 2(a)).  Figure 2(b) shows a representative blot for 4HNE PA in the three study cohorts. In line, the whole blood luminol-dependent CL was increased in obese both with and without MetS and reached the soundest statistical significance versus CTR (*p* < 0.0001) only in the latter group (Figure 2(c)). Plasma coenzyme Q_10_ content (both reduced and oxidized forms) was unaltered versus CTR in obesity with and without MetS (data not shown). Vitamin E (*α*-tocopherol) plasma levels were significantly lower than CTR values (*p* < 0.01) only in the OB w.o.MetS (Figure 2(d)), consistent with the highly significant-versus-baseline elevation of blood CL in the same group (Figure 2(c)). TAC index of plasma antioxidant capacity did not statistically differ among the three groups under study (data not shown). Plasmatic NO_2_^−^/NO_3_^−^ levels were not significantly different among the three study cohorts. Mean ± S.E. values were CTR 20.7 ± 1.05; OB w.MetS 18.2 ± 1.12, and OB w.o.MetS 15.9 ± 1.44.

### 3.4. Erythrocyte Membrane Fatty Acid Profiles

**Figure 3:**
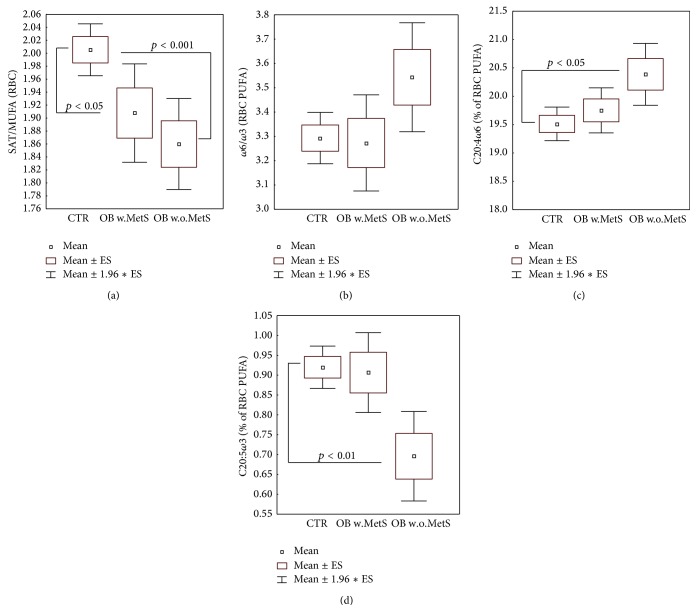
Selected representative parameters describing fatty acid profile of erythrocyte membrane phospholipids: ratio saturated/monounsaturated fatty acids (a), ratio *ω*6/*ω*3 polyunsaturated fatty acids (b), *ω*6-arachidonic acid percent of total FA content of phospholipids (c), and *ω*-3 eicosapentaenoic acid % of total FA content of phospholipids (d), in the groups of obese patients with metabolic syndrome (OB w.MetS, *n* = 45) and without metabolic syndrome (OB w.o.MetS, *n* = 33) and of control healthy subjects (CTR, *n* = 49). Values are represented as mean (□), standard error of the mean (upper and lower limits of the box), and 1.96 × standard error (upper and lower whiskers). Intergroup significant differences (*p*) are indicated in each panel. Abbreviations: SAT (saturated fatty acids); MUFA (monounsaturated fatty acids); PUFA (polyunsaturated fatty acids); C20:4 (arachidonic acid); C20:5 (eicosapentaenoic acid); and RBC (red blood cells). Techniques: gas chromatography-mass spectrometry with selected ion monitoring (a–d).

Figure 3 describes peculiar alterations of the fatty acid profile of RBC membrane phospholipids. In both obese subgroups, a significant increase (*p* < 0.05–0.001) of monounsaturated FA was recorded, expressed as the decreasing ratio SAT/MUFA (Figure 3(a)). As a whole, *ω*-3 FA were not decreased in obese groups, as reported in Figure 3(b), where the ratio *ω*-6/*ω*-3 was not significantly increased versus control group. Nevertheless, Figures 3(c) and 3(d) show that in the OB w.o.MetS patients the percentage compostion of the most abundant *ω*-6 FA, arachidonic acid (C20:4), is significantly raised (*p* < 0.05), whilst the most representative *ω*-3 FA, eicosapentaenoic acid (C20:5), is sharply decreased (*p* < 0.01).

### 3.5. Cytokine and Adipokine Plasma Profiles

**Figure 4:**
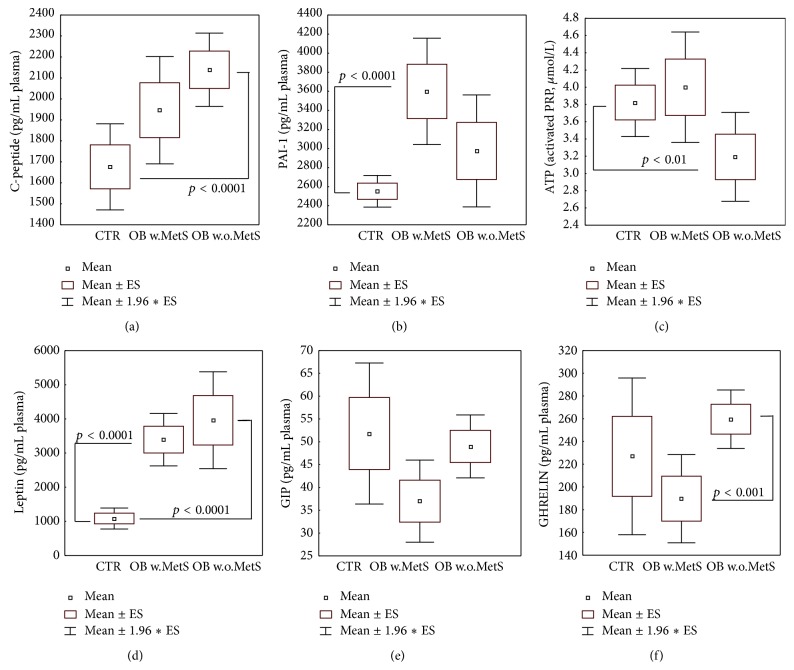
Plasma levels of selected adipokines: C-peptide (a), plasminogen activator inhibitor-1 (b), leptin (d), glucose-dependent insulinotropic polypeptide (e), ghrelin (f), and ATP levels in platelet-enriched plasma (c), in the groups of obese patients with metabolic syndrome (OB w.MetS, *n* = 67) and without metabolic syndrome (OB w.o.MetS, *n* = 45), and control healthy subjects (CTR, *n* = 90). Values are represented as mean (□), standard error of the mean (upper and lower limits of the box), and 1.96 × standard error (upper and lower whiskers). Intergroup significant differences (*p*) are indicated in each panel. Abbreviations: PAI-1 (plasminogen activator inhibitor-1); PRP (platelet-rich plasma); and GIP (glucose-dependent insulinotropic polypeptide). Techniques: multiplexed Bio-Plex Suspension Array System (a-b, d–f), bioluminescence (c).

Of the panel of ten main plasma adipokines measured, four were significantly different in the obesity subgroups as compared to healthy controls. C-peptide levels were highly increased in OB w.o.MetS (*p* < 0.0001) while MetS values did not meet statistical significance requirements (Figure 4(a)). PAI-1 was dramatically higher versus CTR in MetS group (*p* < 0.0001, Figure 4(b)). The increase of ATP levels in plasma activated platelets did not reach statistical significance in the same OB subgroup, whilst resulting to be significantly lower-than-CTR (*p* < 0.01) in OB w.o.MetS (Figure 4(c)), where PAI-1 was not increased. GIP levels were unchanged versus CTR in both obese groups (Figure 4(e)). As expected, leptin levels were elevated in OB w.MetS and OB w.o.MetS versus CTR (*p* < 0.0001), while the anti-inflammatory ghrelin levels, though not showing any significant difference versus CTR, displayed clearcut in-between differences (*p* < 0.001) in the two OB subgroups (Figures 4(d) and 4(f)). No differences among the three patient cohorts were registered for GLP-1, glucagon, insulin, resistin, and visfatin (data not shown).

**Figure 5:**
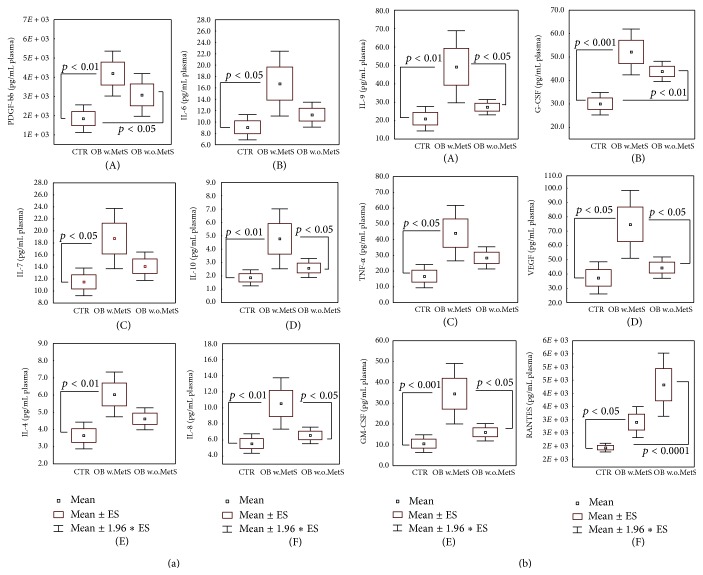
(a) Plasma levels of selected cytokines/chemokines/growth factors: platelet-derived growth factor (A), interleukin 6 (B), interleukin 7 (C), interleukin 10 (D), interleukin 4 (E), and interleukin 8 (F), in the groups of obese patients with metabolic syndrome (OB w.MetS, *n* = 67) and without metabolic syndrome (OB w.o.MetS, *n* = 45) and of control healthy subjects (CTR, *n* = 90). Values are represented as mean (□), standard error of the mean (upper and lower limits of the box), and 1.96 × standard error (upper and lower whiskers). Intergroup significant differences (*p*) are indicated in each panel. Abbreviations: PDGF-bb (platelet-derived growth factor); IL-6 (interleukin 6); IL-7 (interleukin 7); IL-10 (interleukin 10); IL-4 (interleukin 4); and IL-8 (interleukin 8). Techniques: multiplexed Bio-Plex Suspension Array System (A–F). (b) Plasma levels of selected cytokines/chemokines/growth factors: interleukin 9 (A), granulocyte colony stimulating factor (B), tumor necrosis factor-*α* (C), vascular endothelial growth factor (D), granulocyte-macrophage colony stimulating factor (E), regulated on activation, normal T cell expressed and secreted (F), in the groups of obese patients with metabolic syndrome (OB w.MetS, *n* = 67) and without metabolic syndrome (OB w.o.MetS, *n* = 45), and control healthy subjects (CTR, *n* = 90). Values are represented as mean (□), standard error of the mean (upper and lower limits of the box), and 1.96 × standard error (upper and lower whiskers). Intergroup significant differences (*p*) are indicated in each panel. Abbreviations: IL-9 (interleukin 9); G-CSF (granulocyte colony stimulating factor); TNF-alpha (tumor necrosis factor-*α*); VEGF (vascular endothelial growth factor); GM-CSF (granulocyte-macrophage colony stimulating factor); and RANTES (regulated on activation, normal T cell expressed and secreted). Techniques: multiplexed Bio-Plex Suspension Array System (A–F).

The significant results of the multiarray analysis of the 27-plex panel of plasma inflammatory cytokines, chemokines, and growth factors are shown in Figures 5(a) and 5(b). All the twelve proteins shown, that is, PDGF-bb, IL-6, Il-7, Il-10, IL-4, IL-8 (Figure 5(a) (A)–(F)), IL-9, G-CFS, TNF-*α*, VEGF, GM-CFS, and RANTES (Figure 5(b) (A)–(F)), were significantly higher (*p* < 0.05–*p* < 0.001) in the plasma of OB w.MetS as compared to CTR and also to OB w.o.MetS groups. In all cases, plasma levels in OB w.o.MetS were not statistically different versus CTR, with the exception of a significant increase (*p* < 0.0001) for PDGF-bb (Figure 5(a) (A)), G-CFS, and RANTES (Figure 5(b) (B) and (F)).

## 4. Discussion

The primary goal of the present clinical laboratory trial was to evaluate the patterns of oxidative stress, adipokines, and inflammatory factors in two groups of obese people with similar clinical features of obesity but with different metabolic status. One group was clinically and biochemically defined as having a MetS, and the other did not have as yet all metabolic derangements characteristic for “unhealthy” obesity (Table 1). The table shows that patients of both groups were overweighted (BMI), had central type of obesity with fat accumulation around the waist (Circumference), and similar highly increased versus normal ratios of fat/lean tissue mass. Nevertheless, the obese groups under investigation differed substantially in the widely accepted metabolic markers of MetS, such as dyslipidemia, insulin resistance, and borderline fasting glucose levels attributed to prediabetes [12, 15, 16, 21]. Majority of the OB+MetS group patients suffered from arterial hypertension (data not shown). The large female sex prevalence is in fact a common occurrence in obesity facilities in Italy. The group of males (12 out of 112 obese subjects included in the study) did not show peculiar differences in the metabolic and immunologic parameters with the females and therefore was included in the general evaluation.

There is a continuous debate over the causes and possible mechanisms of transition from benign obesity to obesity aggravated by serious metabolic disorders (MetS) bearing high incidence of cardiovascular complications and type II diabetes [9–11, 16, 19–25, 57]. Here, we attempted to elucidate an impact of redox and adipokine homeostasis players and definite inflammatory factors to the effective metabolic differences in benign obesity and unhealthy obesity complicated with MetS. It was not surprising for us that a redox imbalance in favour of oxidative stress with suppressed glutathione and GST (Figure 1), higher-than-normal levels of 4-HNE protein adducts, and luminol-dependent chemiluminescence (Figure 2) was observed in both groups of obese people independently on the presence of metabolic abnormalities. In accordance with recent literature data, 4-HNE has been found in greater-than-normal amounts in obese people [58, 59]. The aldehyde is a final product of lipid peroxidation with diabetogenic potential through induction of insulin resistance and capacity to disrupt adipogenic functions [58–60]. GST protein is sensitive to 4-HNE adduct formation and therefore may be partially inactivated by 4-HNE excess [61] in the obese. In a loop, enzyme inactivation could sustain the increased levels of 4-HNE observed, since GST enzyme together with glutathione inactivates aldehydes [60, 62, 63]. Luminol-dependent chemiluminescence (CL) reflects the capacity of ROS production by circulating granulocytes and monocytes [64]. Since these circulating phagocytes are primed by oxidized low-density lipoproteins (oxLDL) [65–67] to release large amounts of ROS, it appears to be logic that ROS production in obese subjects in general is increased. A vicious cycle is formed when LDL are oxidized by ROS, while oxLDL induce ROS overproduction.

The combination of low level vitamin E, activated SOD, and inhibited CAT in the group with uncomplicated obesity (Figures 1 and 2) allowed us to hypothesize that the high levels of hydrogen peroxide formed in the reaction of superoxide anion-radicals dismutation by SOD (2O_2_^−^ + 2H^+^ + 2H_2_O → 2H_2_O_2_) persist due to inadequate activity of CAT, a hydrogen peroxide decomposing enzyme (2H_2_O_2_ → 2H_2_O + O_2_). Catalase activity is usually inhibited by excess H_2_O_2_, as well as by 4-HNE, which forms aldehyde-protein adducts with the enzyme, thus inactivating its active centre [61, 68, 69]. Hydrogen peroxide belongs to stable reactive oxygen species which, in the presence of transition metals, could be easily converted into hydroxyl radicals (H_2_O_2_ + Fe^**+n**^ → 2OH^*∙*^), classical initiators of lipid peroxidation chain reaction. Under these circumstances, *α*-tocopherol plays a classical role of sacrificing antioxidant being consumed in the process of interruption of the chain reaction in lipid compartments [70–72].

Of great importance, GPX is hyperactivated in the group with MetS exclusively (Figure 1). PUFA in the membrane phospholipids or in circulating lipoproteins are subjected to intense enzymatic (12/15 lipoxygenase) or nonenzymatic free radical-driven lipid peroxidation, under certain pathological conditions such as inflammation, obesity, or atherosclerosis [73]. Both types of lipid oxidation result in the formation of highly reactive lipid hydroperoxides (LOOH), proinflammatory leukotrienes among them, which are neutralised by GPX (mainly, by isoform Gpx4) [74], GST [75], peroxireductase VI, and aldo-keto-reductases [73]. LOOH [73, 76] and small end-products of lipid peroxidation such as 4-HNE and other aldehydes (malonyldialdehyde, acrolein, etc.) transported by activated granulocytes/monocytes may facilitate and maintain generalised inflammation [77–79]. Of interest, successful anti-inflammatory therapy of psoriasis patients with TNF-*α* antibodies led to suppression of GPX activity, while in nonresponders to the therapy, GPX activity was further stimulated [80].

In addition to the possible pathogenic role(s) of oxidative processes in lipids, a significant elevation of monounsaturated fatty acids (MUFA) in erythrocyte membranes assessed by the decreased ratio of saturated FA/MUFA was shown for both experimental obese groups, while increased content of *ω*-6 versus decreased *ω*-3 membrane-bound FA was found in uncomplicated obesity group only (Figure 3). Membrane-bound MUFA are considered as a strong proinflammatory factor in obesity because their biosynthesis is associated with hyperactivated enzyme stearoyl-delta9-desaturase [81, 82]. The combination of two factors, highly increased MUFA and PAI-1 (Figure 4), observed in the Ob+MetS group is associated with the increased risk of cardiovascular diseases, high blood pressure [83], and the elevated coagulation rates in MetS [84].

Adipose tissue was considered as a mere energy storage until the first adipokine, a bioactive product synthesised within and released from adipose tissue, was identified in 1994 [27]. This very first adipokine was leptin, levels of which are highly increased in obese people (Figure 4 and [85, 86]). Adipokines control distinct essential physiological processes, such as appetite and satiety, energy metabolism, endothelial function, hemostasis, blood pressure, insulin secretion and sensitivity, adipogenesis, adipocyte functions, and fat distribution [29]. Among the more than 600 putative adipokines, there are numerous proinflammatory mediators, including interleukins, tumor necrosis factor- (TNF-) alpha, chemokines, and growth factors [87, 88]. A distinct adipokine pattern associated with body weight includes insulin, triglycerides, leptin, PAI-1, chemerin, MCP-1, and retinol-binding-protein-4 (RBP-4) [86]. Some adipokines, such as adiponectin, leptin, chemerin, visfarin, and PAI-1, are produced exclusively by adipose tissue, while others, tumor necrosis factor- (TNF-) alpha, interleukins, chemokines, and growth factors, could be produced by circulating blood leukocytes, tissue macrophages, keratinocytes, and other cells. Therefore, blood levels of these factors reflect not only their release from adipose tissue but also a significant contribution of circulating leukocytes, cells-effectors of inflammatory responses in the organism, and endothelial cells. Out of all pure adipokines, plasma levels of which were measured in the study; exclusively leptin, a marker of obesity, exhibited higher-than-normal values in both obese groups (Figure 4), while visfatin and resistin remained within the normal range (data not shown). Another adipokine PAI-1 produced in visceral and not in subcutaneous adipose tissue was overexpressed in patients with MetS; its high levels are connected with high risk of thrombosis [84]. A clearcut proinflammatory pattern of cytokines (IL-4, IL-6, IL-7, and IL-9), chemokines (IL-8), growth factors (VEGF, PDGF, G-CSF, GV-CSF), and TNF-*α* was found in obese subjects with MetS. As an adaptive reaction, the expression of anti-inflammatory IL-10 was also increased (Figures 5(a) and 5(b)). These findings corresponded to an assumption that MetS is a pathology characterised by generalised chronic inflammation [2, 4, 31, 89–91].

Of major relevance is the contribution of TNF-*α* to metabolic syndrome. TNF-*α* expression was found to be increased in the adipocytes of obese animals, and its neutralisation by a TNF-*α*-specific soluble antibody led to the improvement of insulin sensitivity [92]. These observations were then confirmed in obese and diabetic humans [93]. Noteworthy, the major source of TNF-*α* as well as of multiple cytokines and chemokines in obesity are phagocytes, not adipocytes [94]. The therapy with anti-TNF-*α* antibodies resulted in attenuation of MetS symptoms in general [95] and decrease of GPX activity to neutralise LOOH and toxic aldehydes [80]. All these inflammation-related cytokines could be overproduced by circulating leukocytes primed with LDL and free fatty acids (FFA) [67].

Of note, obesity itself was not connected to abnormal plasma mediators of inflammation with the exception of RANTES, which was remarkably increased in the OB group. We assume that RANTES could be a key molecular target to interrupt an undesired transition to MetS in obese people. Regulation of leukocyte activation and migration into tissues by chemokines like RANTES and similar monocyte chemotactic protein-1 (MCP-1) are recognised as important factors in the induction of acute and chronic inflammation [96]. RANTES has been shown to stimulate T-lymphocyte extravasation as well as monocyte oxidative metabolism and recruitment to lungs, kidney, liver, skin, and other tissues [97]. The inhibition of RANTES and MCP-1 with corresponding antibodies or low molecular weight antagonists has been suggested and successfully applied in many pathologies accompanied by chronic inflammation [98]. RANTES may represent a valuable target for the prevention of MetS setting as well as for diagnostic purposes.

## 5. Conclusions

On the grounds of the results obtained, we hypothesised that as a primary step of the transition from the benign obesity to obesity complicated with MetS there could be a long-lasting priming of circulating phagocytes by excessive amount of oxLDL. The primed granulocytes and monocytes produce large amount of ROS and redox-dependent proinflammatory cytokines. Primed leukocytes in benign obesity overexpress chemokines like RANTES, thus prompting leukocyte migration into tissues, for example, in adipose tissue, pancreas, muscles, and liver, which are target organs for the initiation and maintenance of metabolic derangements characteristic for MetS (the first loop of transition). Subsequently, ROS-mediated formation of LOOH and toxic end-products of lipid peroxidation occurs. These highly reactive products induce adipogenesis and fat production by adipocytes [65–67, 99–101], which creates a second self-sustained pathological loop of increased fat production and accumulation → increased fatty acid oxidation → chronic proinflammatory pattern formation → redox-dependent derangement of antioxidant and detoxifying systems → redox-dependent derangement of insulin sensitivity → redox-dependent dyslipidemia → MetS. Upregulated GPX could be an early pathogenic marker of MetS setting, as a unique defense against hydrogen peroxides and their metabolites with a pathogenic role for MetS in the obese.

## Figures and Tables

**Table 1:** Biometric and clinical chemistry parameters (mean ± SEM) of the two patient groups under study: obese with metabolic syndrome (OB w.MetS) and obese without metabolic syndrome (OB w.o.MetS).

Parameter	OB w.MetS (*n* = 67, 59 F/8 M)	OB w.o.MetS (*n* = 45, 41 F/4 M)
BMI (kg/m^2^)	41.8 ± 1.5	38.3 ± 1.3^∗^
WAIST CIRCUMFERENCE (cm)	121.7 ± 2.3	115.0 ± 2.7
FAT MASS (%)	42.2 ± 0.9	42.7 ± 1.3
LEAN MASS (%)	57.8 ± 0.9	57.3 ± 1.3
PLASMA TOTAL CHOL (mg/dL)	211.0 ± 7.8	190.8 ± 6.5
PLASMA LDL (mg/dL)	135.2 ± 5.8	119.7 ± 6.8
PLASMA HDL (mg/dL)	42.5 ± 1.3	55.2 ± 2.1^#^
PLASMA TG (mg/dL)	190.8 ± 15.4	81.9 ± 4.7^#^
PLASMA TOTAL CHOL/HDL ratio	5.1 ± 0.2	3.59 ± 0.2^#^
PLASMA TG/HDL ratio	4.71 ± 0.5	1.57 ± 0.1^#^
FASTING GLUCOSE (mg/dL)	104.8 ± 3.7	86.7 ± 1.9^§^
FASTING INSULIN (*μ*U/mL)	34.3 ± 3.6	21.5 ± 2.4^§^
CRP (mg/dL)	1.1 ± 0.2	0.7 ± 0.1
ALP (IU/L)	95.2 ± 7.0	76.3 ± 6.4
AST (IU/L)	27.4 ± 2.6	18.1 ± 1.1^§^
ALT (IU/L)	43.9 ± 6.5	39.2 ± 16.2
gammaGT (IU/L)	42.9 ± 7.0	20.6 ± 4.3^#^

^#^ *p* < 0.0001; ^§^ *p* < 0.001; ^∗^ *p* < 0.05.

Abbreviations: BMI (body mass index); CHOL (cholesterol); LDL (low density lipoproteins); HDL (high density lipoproteins); TG (triglycerides); CRP (C-reactive protein); ALP (alkaline phosphatase), AST (aspartate aminotransferase), ALT (alanine aminotransferase), gamma GT (gamma glutamyltransferase); U (units); IU (international units).
